# Fibrinogen as a Pleiotropic Protein Causing Human Diseases: The Mutational Burden of Aα, Bβ, and γ Chains

**DOI:** 10.3390/ijms18122711

**Published:** 2017-12-14

**Authors:** Elvezia Maria Paraboschi, Stefano Duga, Rosanna Asselta

**Affiliations:** 1 Department of Biomedical Sciences, Humanitas University, Via Rita Levi Montalcini 4, 20090 Pieve Emanuele, Milan, Italy; elvezia_maria.paraboschi@hunimed.eu (E.M.P.); stefano.duga@hunimed.eu (S.D.); 2 Humanitas Clinical and Research Center, Via Manzoni 56, 20089 Rozzano, Milan, Italy

**Keywords:** fibrinogen, afibrinogenemia, hypofibrinogenemia, dysfibrinogenemia, hepatic fibrinogen storage disease, hereditary renal amyloidosis, *FGA*, *FGB*, *FGG*, exome-based epidemiology

## Abstract

Fibrinogen is a highly pleiotropic protein that is involved in the final step of the coagulation cascade, wound healing, inflammation, and angiogenesis. Heterozygous mutations in Aα, Bβ, or γ fibrinogen-chain genes (*FGA*, *FGB*, *FGG*) have been described as being responsible for fibrinogen deficiencies (hypofibrinogenemia, hypo-dysfibrinogenemia, dysfibrinogenemia) and for more rare conditions, such as fibrinogen storage disease and hereditary renal amyloidosis. Instead, biallelic mutations have been associated with afibrinogenemia/severe hypofibrinogenemia, i.e., the severest forms of fibrinogen deficiency, affecting approximately 1–2 cases per million people. However, the “true” prevalence for these conditions on a global scale is currently not available. Here, we defined the mutational burden of the *FGA*, *FGB*, and *FGG* genes, and estimated the prevalence of inherited fibrinogen disorders through a systematic analysis of exome/genome data from ~140,000 individuals belonging to the genome Aggregation Database. Our analysis showed that the world-wide prevalence for recessively-inherited fibrinogen deficiencies could be 10-fold higher than that reported so far (prevalence rates vary from 1 in 10^6^ in East Asians to 24.5 in 10^6^ in non-Finnish Europeans). The global prevalence for autosomal-dominant fibrinogen disorders was estimated to be ~11 in 1000 individuals, with heterozygous carriers present at a frequency varying from 3 every 1000 individuals in Finns, to 1–2 every 100 individuals among non-Finnish Europeans and Africans/African Americans. Our analysis also allowed for the identification of recurrent (i.e., *FGG*-p.Ala108Gly, *FGG*-Thr47Ile) or ethnic-specific mutations (e.g., *FGB*-p.Gly103Arg in Admixed Americans, *FGG*-p.Ser245Phe in Africans/African Americans).

## 1. Introduction

Fibrinogen is a 340-kDa glycoprotein that plays a crucial role in the hemostatic cascade, being the substrate for fibrin clot formation and the support for platelet aggregation [1,2,3]. It is also essential for several other biological functions, such as wound healing, inflammation, and angiogenesis [4,5,6].

Into the circulation, thrombin cleaves fibrinopeptides A and B to convert fibrinogen into fibrin, which spontaneously polymerizes, and thus forms double-stranded protofibrils, which, in turn, assemble into fibers, ultimately leading to the fibrin clot [7]. In hepatocytes—the primary site of fibrinogen biosynthesis—the molecule is rapidly assembled in the endoplasmic reticulum (ER), and secreted as a hexamer composed of two sets of three homologous polypeptide chains (called Aα, Bβ, and γ) [8,9,10,11]. These are encoded by paralogous genes (*FGA*, *FGB*, and *FGG*), which are clustered in a 50-kb region on chromosome 4 (4q31.3–q32.1) [12].

Monoallelic and biallelic mutations in *FGA*, *FGB*, and *FGG* genes are associated with different inherited conditions, reflecting the pleiotropic function of the fibrinogen protein [13]. Congenital fibrinogen defects are conventionally classified on the basis of plasma concentration as quantitative (type I) and qualitative (type II) deficiencies [14,15,16]. Quantitative deficiencies include afibrinogenemia/severe hypofibrinogenemia (Online Mendelian Inheritance in Man (OMIM) #202400; [17]) and hypofibrinogenemia (OMIM +134820, *134830, *134850), which are characterized by the lack/extremely low or by reduced amounts of immunoreactive fibrinogen (<150–160 mg/dL), leading to hemorrhagic manifestations, which can vary from very mild to life threatening. Qualitative deficiencies comprise dysfibrinogenemia (OMIM +134820, *134830, *134850) and hypo-dysfibrinogenemia, and are characterized by a discrepancy between antigen levels and functional (abnormally low) activity. Patients that are diagnosed with these conditions are either asymptomatic, or can suffer from bleeding symptoms, thrombophilia, or even both [15].

Hypofibrinogenemia and afibrinogenemia have long been considered as different clinical entities. Indeed, they represent the phenotypic expression of the same quantitative trait (i.e., diminished plasma fibrinogen level), which is determined, respectively, by heterozygous or homozygous/combined heterozygous mutations that are affecting one of the fibrinogen genes. As for dysfibrinogenemias and hypodysfibrinogenemias, these disorders are usually inherited as an autosomal dominant trait: they are caused by a single genetic defect ultimately affecting a functional property of the fibrinogen protein, such as the release (impaired or delayed) of fibrinopeptides A and B, defective polymerization, crosslinking, or thrombin binding, as well as delayed plasmin digestion [16].

Rare hypofibrinogenemic patients can present with liver disease due to the accumulation of mutant fibrinogens within hepatocytes. This condition is called fibrinogen storage disease (FSD), and is generally caused by heterozygous mutations, leading to an impaired secretion of the abnormal fibrinogen, which however maintains its capacity for polymerization and spontaneously aggregates in hepatocellular ER. In the vast majority of cases, mutations leading to FSD are missense variants that are located in a defined region of the C-terminal γ chain (residues 284–375) [18,19]. FSD-causing mutation carriers show a great variability in the severity of liver injury, going from the lack of symptoms to severe liver fibrosis/cirrhosis; more severe manifestations can be secondary to xenobiotic intake (e.g., estrogen therapy, alcohol abuse), to viral infections, or even to cancer [18,20].

Heterozygous mutations, which are affecting a small region of the C-terminal portion of the Aα chain, have been described in patients with hereditary renal amyloidosis (HRA) (OMIM +134820). These genetic defects are associated with a mild decrease in fibrinogen levels and are supposed to destabilize the native fold of circulating Aα chain degradation peptides, so that they spontaneously aggregate into amyloid fibrils, prevalently in the glomeruli of the kidneys, and, to a lesser extent, in heart muscle, spleen, and liver. Fibrinogen amyloidosis is the most common form of HRA, and clinical symptoms include hypertension, proteinuria, and azotemia [21].

The frequency of congenital fibrinogen disorders in the general population is very low. International registries, such as those from the United States, Italy, Iran, and the United Kingdom, suggest that afibrinogenemia is one of the rarest among rare bleeding disorders, with only 1–2 cases per million people [22]; however, these registries lack prospective and systematic evaluations that could lead to incidence/prevalence determination. In addition, “true” incidence/prevalence estimates for a-, hypo-, and dysfibrinogenemia are made difficult, because many patients are asymptomatic [15,22].

With these premises, we here defined the global mutational landscape of *FGA*, *FGB*, and *FGG*, and tried to determine ethnic-specific prevalence of inherited fibrinogen disorders, by analyzing exome and genome data from almost 140,000 individuals available through the publicly available genome Aggregation Database (gnomAD) resource [23,24].

## 2. Results

### 2.1. Global Mutational Spectrum of the Fibrinogen Gene Cluster Using Population-Based Exome- and Genome-Sequencing Data

High-quality variants were collected from the gnomAD database in subjects of different ethnicities, i.e., Africans and African Americans (12,020 subjects), admixed Americans (17,210), Ashkenazi Jewish (5076), East Asians (9435), Finnish (12,897), non-Finnish Europeans (63,369), South Asians (15,391), and other ethnicities (population not assigned; 3234 subjects).

Among the variants reported in the repository with high confidence, we retrieved all of the null mutations (frameshifts, nonsense, and splicing variants affecting the first two and the last two intronic nucleotides), the non-synonymous variants annotated as damaging by seven out of seven prediction programs, as well as splicing defects mapping in intronic positions −3 and +3/+6 and predicted as disrupting by three out of three prediction algorithms.

We found a total of 1524 heterozygous variants (287 in *FGA*, 233 in *FGB*, 1004 in *FGG*) in a maximum of 277,080 different alleles, corresponding to a total of 189 unique mutations (79 in *FGA*, 53 in *FGB*, 57 in *FGG*) (Table 1). Among these unique mutations, 56 (29.6%) have already been reported in fibrinogen-disorder related databases. As for the 133 mutations that were not described in the literature, in Supplementary Table S1 we listed all of the null frameshift/nonsense mutations, whereas in Supplementary Table S2, we reported all of the missense/splicing/inframe-indels mutations consistently annotated as damaging by prediction programs.

**Figure 1:**
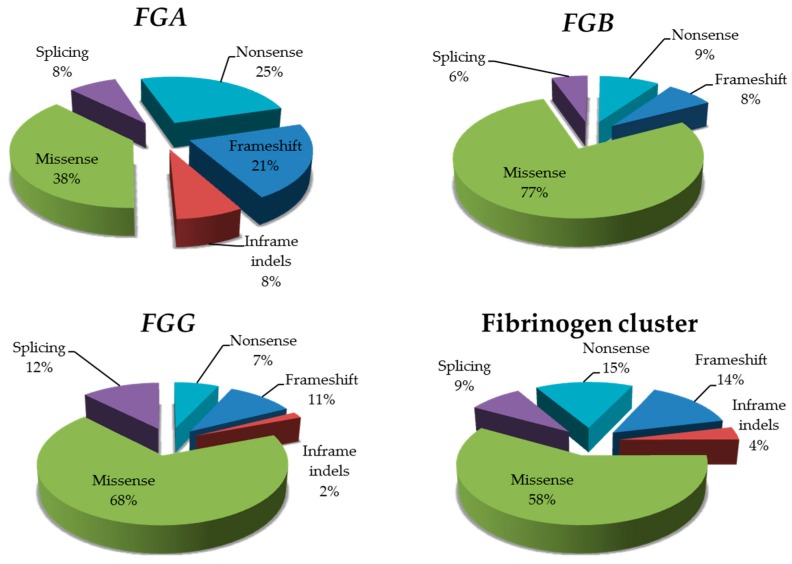
Distribution of point mutations in the fibrinogen genes. Pie-charts show the distribution of the different types of point mutations identified in each fibrinogen gene; an overall count is also displayed for the whole fibrinogen cluster.

The distributions of the different types of mutations in the three genes are summarized in Figure 1: distributions were not significantly different from those reported in the Human Gene Mutation Database (HGMD) (Fisher exact test, 2-tailed *p* values 0.32, 0.69, and 0.83, for *FGA*, *FGB*, *FGG*, respectively).

### 2.2. Carrier Rates of Recessively-Inherited Fibrinogen Deficiencies Using Population-Based Exome- and Genome-Sequencing Data

At first, we performed prevalence calculations for the autosomal-recessive fibrinogen deficiency forms by considering separately the *FGA*, *FGB*, and *FGG* genes (Table 2). In general, our analysis revealed prevalence data profoundly different among the three genes, as well as among different populations. In fact, global prevalence rates increase from 0.31 per 10^6^ individuals for the *FGA* gene, to 0.69 per 10^6^ individuals for *FGB*, up to the remarkable prevalence of 9.54 per 10^6^ individuals for the *FGG* gene. Autosomal-recessive fibrinogen deficiency appears to be virtually absent in Ashkenazi Jews (no mutations found in *FGA* and *FGB*; prevalence for *FGG* of 1.64 per 10^6^ individuals), whereas, non-Finnish Europeans have a predicted exceptional rate of 23.05 per 10^6^ individuals for the sole *FGG* gene.

Then, we performed prevalence calculations collapsing together the rates relative to the three genes. Table 3 shows the world-wide prevalence rate, as well as the prevalence calculated for the different populations. In particular, the world-wide prevalence was ~10 fold higher than that reported so far (i.e., 1 in 10^5^ rather than 1 in 10^6^); Europeans showed the highest prevalence (~2.5 in 10^5^), whereas Ashkenazi Jews and East Asians the lowest (~1.6 in 10^6^ and ~1.1 in 10^6^, respectively).

Finally, the interrogation of the gnomAD database allowed for us to point out recurrent and ethnic-specific mutations (Table 4). Among them, two mutations that were located in *FGG* were extremely frequent in the analyzed populations, i.e., p.Ala108Gly and p.Thr47Ile. Both of the mutations have been described in the literature as being causative of hypofibrinogenemia and associated with a mild bleeding tendency [25,26,27]. In particular, the p.Ala108Gly variant (also known as Ala82Gly, fibrinogen Dunedin) accounts for the totality of mutated alleles in Ashkenazi Jews, and represents >70% of all mutated alleles among Europeans (for a total of 29 heterozygous individuals among 25,790 Finns, and 456 heterozygous individuals among 126,630 non-Finnish Europeans).

### 2.3. Global Carrier Rates of Autosomal-Dominant Fibrinogen Disorders Using Population-Based Exome- and Genome-Sequencing Data

Apart from afibrinogenemia/severe hypofibrinogenemia, all of the fibrinogen disorders are transmitted in an autosomal dominant fashion, and are characterized by the presence of one causative mutation involving any of the three fibrinogen-chain genes. Hence, we performed a global prevalence calculation for such disorders by clumping together the contribution of *FGA*, *FGB*, and *FGG*.

Our analysis estimated that heterozygous subjects should be present in the general world-wide population, at a frequency of ~1 every 100 individuals (range: 0.3 to 1.5 every 100 individuals), suggesting that fibrinogen-related disorders represents a far more frequent condition in the population than believed so far (Table 5).

Finally, we specifically searched the gnomAD repository for the presence of mutations that were known to be frequently found in patients affected by dysfibrinogenemia [28], FSD [18], or HRA [29] (Table 6). No FSD-causing mutation was found in database, and only eight heterozygous individuals were carrier of the well-known mutation hotspots *FGA*-p.Arg35Cys/His or *FGG*-p.Arg301His (these mutations account for 85% of all known cases of congenital dysfibrinogenemia) [28]. As for HRA-causing mutations, we found just one heterozygous carrier of the *FGA*-p.Glu545Val variant (i.e., the most common HRA variant) [29], but we observed a total of 29 carriers of p.Glu545Lys, i.e., a mutation less-frequently associated with HRA, but involving the same *FGA* p.Glu545 residue. Interestingly, 19 of 29 p.Glu545Lys carriers were of Ashkenazi Jewish origin, so that the calculated prevalence of heterozygotes for this mutation in this population reaches the remarkable rate of 3.74 in 10^3^ individuals.

## 3. Discussion

With the exception of the involvement of some peculiar molecular mechanism, such as uniparental isodisomy of the entire chromosome 4 [30], the genetic bases of fibrinogen disorders are invariably constituted by homozygous/heterozygous mutations within the fibrinogen gene cluster. Here, we took advantage of exome and genome data of ~140,000 individuals to estimate the prevalence of recessively-inherited fibrinogen deficiency, as well as the collective prevalence of all the dominantly-inherited fibrinogen disorders. Our estimates indicated that: (i) the world-wide prevalence for recessively-inherited fibrinogen deficiencies could be 10-fold higher than that reported so far; (ii) prevalence among different populations seems to be extremely different (ranging from 1 in 10^6^ in East Asians up to 24.5 in 10^6^ in non-Finnish Europeans); and, (iii) heterozygous carriers of mutations in the fibrinogen cluster (i.e., individuals possibly at risk to develop a form of fibrinogen disorder, as well as asymptomatic/undiagnosed subjects) should be present in the general world-wide population at a frequency of ~1 every 100 individuals.

Notably, we have to acknowledge that our estimates suffer from some important limitations. First, our prevalence calculations relied on the use of prediction programs aimed at evaluating the deleteriousness of missense/splicing mutations with unknown biological significance. In this respect, we have to notice that, although these algorithms can present limitations [31,32], their use currently represents a standard approach, especially if researchers have to deal with data from large-scale sequencing projects. Indeed, it has been demonstrated that different methods can individually show a limited overall predictive value, which, however, increases significantly when considering only concordant outputs from different software (e.g., it has been calculated an encouraging predictive value of ~90% when taking into account concordant results from four different prediction methods) [31]. We hence based our prevalence calculations on the use of seven different prediction software for missense variants, and of three programs for splicing variants. This choice proved to be quite “conservative”: for instance, excluding from prevalence calculations all of the variants that were never reported in fibrinogen-related databases, we observed a global prevalence rates for recessively-inherited fibrinogen deficiencies of 6.8 in 10^6^ individuals (0.033 in 10^6^ individuals for the *FGA* gene, 0.16 in 10^6^ for *FGB*, 6.68 in 10^6^ for *FGG*). Importantly, the good performance of our in-silico approach is also testified by predictions performed on fibrinogen missense variants reported as associated with fibrinogen disorders in the databases: we observed an overall prediction rate for the fibrinogen cluster of 78% (if considering all together concordant predictions from seven of seven and six of seven algorithms). The only exception pertained the nine *FGA* mutations that were described as cause of amyloidosis: none of them was predicted as damaging neither by 7 of 7 nor by 6 of 7 algorithms. This was probably because all these variants involve a peculiar trait of the Aα chain, i.e., the C-terminal unstructured tail of the protein.

Second, we could in theory have underestimated the total number of causative mutations for systematic bias still characterizing exome-sequencing data. For instance, promoters and intronic regions are not included by design in exome sequencing, so that variants located in such regions can easily go undetected. Insertions and deletions are not always correctly recognized by variant-calling programs, so they can go unnoticed as well. More importantly, gross deletions, and rearrangements may be not detected at all. This represents a substantial problem in the calculation of prevalence for fibrinogen deficiencies, since one of the most recurrent mutation for these disorders is the well-known 11-kb deletion, eliminating the majority of the *FGA* gene [16,33]. This deletion has been reported in at least eight afibrinogenemic patients [34], and, together with other two gross deletions that are described in the *FGA* gene (i.e., a 15-kb and a 4.1-kb deletion) [30,35], account for ~9% of all cases of afibrinogenemia characterized by mutations in *FGA* [34].

The high prevalence rates that we estimated from the gnomAD repository are indeed not completely unexpected. The most recent World Federation of Hemophilia (WFH) annual global survey, which was conducted in 2015, found that inherited fibrinogen deficiencies on a global scale account for 1777 of 304,362 inherited bleeding disorders (0.6%) [36], confirming fibrinogen deficiencies as quite rare disorders, and approximately 85 times less common than hemophilia A (these data are based on questionnaires sent to national hemophilia associations linked with the Federation). However, digging deeper in the WFH data, it becomes clear that prevalence data on fibrinogen deficiencies are somehow underestimated, at least in some populations. For instance, though it is not clear if data reported concern autosomal-recessive, autosomal-dominant, or both forms of the fibrinogen deficiency, it is possible to calculate exceptional prevalence rates of 8.7 and 14.3 per million people for the United Kingdom and the Slovak Republic, respectively. Concerning Italy, a country with a population that is comparable to that of the United Kingdom (~60 million people), no data are reported on fibrinogen deficiencies in the WFH 2015 survey. However, in the “Human fibrinogen database”, a total of 104 fibrinogen-deficient Italian cases are described (54 coming from our center). This figure alone is sufficient to suggest higher prevalence rates for fibrinogen-related disorders than those reported so far, especially if considering that the database has a clear bias towards published data. To have a better idea of the Italian situation, we took advantage of our whole-exome dataset [37], which was composed of exomes of 1750 healthy controls (80% males, age < 45 years, no history of thromboembolic disease). Sequence coverage of fibrinogen cluster was optimal in all individuals, and allowed us to retrieve a total of 24 variants, corresponding to eight different mutations (one frameshift mutation and seven missense damaging variants; distribution: one variant in *FGA*, two in *FGB*, 5 in *FGG*). Once again, the burden of mutations affecting the *FGG* gene appears to be the highest, with just 1 mutation (p.Ala108Gly) considerably driving the prevalence rates for recessively-inherited fibrinogen deficiencies (0.08 per 10^6^ individuals for the *FGA* gene, 0.33 per 10^6^ individuals for *FGB*, and an exceptional 36 per 10^6^ individuals for the *FGG* gene) (Supplementary Table S3). Interestingly, the “driving effect” that was exerted by the p.Ala108Gly mutation could also be at the basis of the marked differences in prevalence rates that were observed in different populations (from 1 in 10^6^ in East Asians to 24.5 in 10^6^ in non-Finnish Europeans). In particular, Ivaskevicius and colleagues [38] reported that the p.Ala108Gly mutation is associated with a specific haplotype, hence denoting a single, ancestral event (founder effect). Given the observed frequencies of the mutation in the different ethnic groups (Table 4), one could speculate that the ancestral mutation event could have originated in Africans (before the major divergence of non-African populations), and that, subsequently, the mutation could have spread towards Europe and Asia following human migrations [39]. However, with the last divergence between South and East Asians, it is conceivable that the p.Ala108Gly mutation could have not reached the Far East.

The issue related to the fundamental contribution of the p.Ala108Gly mutation to prevalence estimates should be carefully kept in mind. In fact, if we do not consider this variant at all, the prevalence rates for recessively-inherited fibrinogen disorders would dramatically drop (on a global scale prevalence would be 3.2 per million people), with highest values that would be registered in Africans/Africans Americans (i.e., 4 per million people) and the lowest in Ashkenazi Jews (for this population, recessively-inherited fibrinogen deficiencies would be virtually absent). The same dramatic drop would be registered for autosomal-dominant fibrinogen disorders (Supplementary Table S4). The p.Ala108Gly mutation (legacy name γAla82Gly) was repeatedly reported as being associated with moderately-decreased fibrinogen levels and with mild bleeding tendency [25,26,34]. In addition, in a recent meta-analysis aimed at identifying loci for fibrinogen concentration, the p.Ala108Gly allele clearly emerged among the strongest predictors of decreased fibrinogen levels (β = −0.2179; *p* = 4.0 × 10^−82^) [40]. Importantly, Ivaskevicius and colleagues [38], by screening 616 blood donors, already observed the p.Ala108Gly as a common *FGG* variant in Caucasians, thus calculating an allele frequency of 0.0032 and a frequency for homozygous individuals of 1 in 95,000. These data well reconcile with those calculated using the gnomAD database.

Our data raise the problem of why such remarkably high prevalence can be calculated for recessively-inherited fibrinogen deficiencies. The most likely explanation could rely on the relatively-low fibrinogen levels that are associated with the highly-prevalent p.Ala108Gly mutation, and the consequent mild/absent symptomatology characterizing many carrier/homozygous individuals (which can go unnoticed) [38]. Alternatively, we can hypothesize two additional explanations.

A first possibility could be that the p.Ala108Gly allele, at the heterozygous state, confers a selective advantage, and thus, has spread throughout the gene pool. This hypothesis strongly emerges among other genetic explanations, such as a possible genetic drift (which can be excluded due to the presence of the p.Ala108Gly allele world-wide), a potential transmission distortion (which can be left out since the Hardy-Weinberg equilibrium is perfectly respected), or a high mutation rate. This last possibility can be discounted on the basis of the above-mentioned observation that the p.Ala108Gly mutation is associated with a founder effect [38]. It remains to understand why a pro-hemorrhagic allele could represent a selective advantage; in this respect, it is worth noticing that a high fibrinogen concentration has long been recognized as a strong and established predictor of cardiovascular disease outcomes (myocardial infarction, stroke, venous thromboembolism), autoimmune disorders with an inflammatory component, as well as cancer [5,41,42,43,44]. However, most of these phenotypes are late onset and therefore are predicted to have a limited effect on natural selection; moreover, the heterozygous advantage hypothesized to explain the frequency of the factor V Leiden mutation points to the opposite direction, being related to moderate hypercoagulability [45].

Conversely, it could be plausible that we do not detect in the general population the high rate of predicted homozygous/compound heterozygous individuals because of problems that are associated with pregnancy (defects in fetal implantation in afibrinogenemic/hypofibrinogenemic women and/or embryos). It is indeed well recognized that fibrinogen has a critical role in maintaining pregnancy: at six weeks of gestation, maternal endothelial cells are replaced by cytotrophoblasts, starting a remodeling process of vessels that involves an active bleeding near the cytotrophoblastic shell, followed by the formation of the Nitabuch’s layer (a fibrinoid layer). This process is highly compromised in patients with quantitative fibrinogen defects, ultimately leading to spontaneous miscarriages [46]. This notion is well supported by different studies analyzing series of fibrinogen-deficient women, all reporting miscarriage as a frequent complication [47,48,49,50]. Further corroborating this observation, a recent study—aimed at elucidating by whole-exome sequencing the genetic etiology of recurrent pregnancy loss—reported the identification of mutations in the *FGA* gene as having a potential role in implantation/pregnancy biology [51]. Hence, when considering the high frequency of heterozygous carriers of fibrinogen defects in the general population, we can hypothesize the fibrinogen cluster as a future “biomarker” to be screened also for recurrent pregnancy loss.

In conclusion, in this work, we have exploited the enormous potential that is provided by public-available repositories to paint a more clear landscape of the genetic burden associated with fibrinogen genes and their related disorders. From our analysis, it clearly emerges that putative disease alleles are much more frequent than expected, a trend already observed for other genes/disorders [52,53]. Caution should be placed to interpret these data, since some of the identified variants could be non-pathogenic and some others could be not fully penetrant. Nonetheless, our analysis represents the first attempt to evaluate the prevalence rates of fibrinogen disorders in populations other than those that are coming from North America and Europe, also indicating the mutations/genes to be prioritized for genetic screenings.

## 4. Materials and Methods

### 4.1. Retrieving Variants from the GnomAD Database

For calculating prevalence of fibrinogen disorders in different ethnic groups, we retrieved data from the gnomAD database (release 2.0) [23,24], which contains exome and genome data on sequence variation in 138,632 unrelated subjects of different ethnicities (123,136 exomes and 15,496 genomes) (Table 7). These individuals are part of disease-specific and population-genetic studies; subjects suffering from pediatric diseases (as well as their first-degree relatives) are not included in the database.

Among the genetic variants that are present in the gnomAD database, we collected and considered as deleterious:

(i) nonsense and frame-shift variants;
(ii) disruptive splice-site variants affecting the first two or last two intronic nucleotides of the splicing site;
(iii) splice-site variants located at intronic positions −3 and +3/+6 predicted as deleterious by three out of three prediction programs. These software were: Human Splicing Finder [54], NetGene2 [55], and Splice Site Prediction by Neural Network [56]. We considered as deleterious sequence variations predicted either to completely abolish the wild-type splicing site or to determine a decrease in the prediction score to less than half of the wild-type counterpart;
(iv) missense variants reported as cause of fibrinogen disorders in the following databases: the Human Fibrinogen Database (release 43) [34,57]; the public release of Human Gene Mutation Database (HGMD) and its professional version (our release dates back to 2014) [58,59]; the Expert Protein Analysis System (ExPASy) [60,61]; and the ClinVar resource [62,63]; and,
(v) missense variants predicted as deleterious by seven out of seven programs. These software, all included in the dbNSFP package, were: SIFT [64], PolyPhen2 (2 software) [65], MutationTaster [66], MutationAssessor [67], Likelihood Ratio Test (LRT) [68], and Functional Analysis Through Hidden Markov Model (FATHMM) [69].

Among variants present in the gnomAD database, we excluded those:

- showing low-confidence level in the variant calling step (as indicated by the gnomAD server);
- being mapped in *FGA* exon 6 and *FGG* exon 10. Variants in such exons affect low-abundance mRNA isoforms of the relevant chain [14], and were never clearly associated with fibrinogen disorders; and,
- being mapped in introns but annotated within alternative transcripts of the fibrinogen chains of unknown significance.

### 4.2. Calculations of Global Prevalence for FGA, FGB, and FGG Genes

We performed prevalence calculations for

- afibrinogenemia/severe hypofibriniogenmia (autosomal recessive trait); since, we expect to have two in-trans mutations affecting the same gene, in this case, we first considered each gene separately, and subsequently we summed the *FGA*, *FGB*, and *FGG* prevalence for estimating the global rate. For this analysis, we excluded all of the mutations that were reported in fibrinogen-related databases, as associated with dysfibrinogenemia, hypodysfibrinogenemia, and amyloidosis; and,
- all the other fibrinogen disorders together (hypofibrinogenemia, dysfibrinogenemia, hypo-dysfibrinogenemia, FSD, and HRA; all autosomal dominant); in this case, we expect just one mutation in any of the three genes, hence a “cumulative” prevalence was calculated when considering the three genes together.

## Figures and Tables

**Table 1:** Mutational landscape of the three fibrinogen genes.

Gene	Type of Mutation	Number of gnomAD Mutations	Number of gnomAD Mutations Already Reported in Fibrinogen Disorder Databases ^1^	
*FGA*	Frameshift	17	5	4a, 1d/h
	Nonsense	20	11	9a, 2a/h
	Splicing	6	2	2a
	Missense	30	10	6d, 1h, 3m
	Inframe indels	6	2	2a
	Total	79	30	
*FGB*	Frameshift	4	0	-
	Nonsense	5	3	2h, 1a/h
	Splicing	3	1	1h
	Missense	41	7	1a, 2d, 4h
	Inframe indels	0	0	-
	Total	53	11	
*FGG*	Frameshift	6	1	1a
	Nonsense	4	1	1a
	Splicing	7	1	1a
	Missense	39	12	8d, 3h, 1d/h
	Inframe indels	1	0	-
	Total	57	15	

^1^ We interrogated: the Human Fibrinogen Database; the Human Gene Mutation Database (HGMD); the Expert Protein Analysis System (ExPASy); and the ClinVar resource (see Section 4). The phenotype associated with already reported mutations is also reported: a = afibrinogenemia, d = dysfibrinogenemia, h = hypofibrinogenemia, m = amyloidosis.

**Table 2:** Estimated prevalence of recessively-inherited fibrinogen deficiencies by ethnicity and gene.

Gene	Population	Total Number of Alleles ^1^	Total Number of Variants ^2^	Collective Frequency of Variants	Heterozygote Frequency	Prevalence in 10^6^ Individuals
*FGA*	All	277,108	154	0.000556	0.00111	0.31
	Africans and African Americans	24,030	9	0.000374	0.000749	0.14
	Admixed Americans	34,414	41	0.00119	0.00238	1.42
	Ashkenazi Jewish	10,148	0	-	-	-
	East Asians	18,868	8	0.000424	0.000848	0.18
	Finnish	25,794	0	-	-	-
	Europeans (not Finnish)	126,628	67	0.000529	0.00106	0.28
	South Asians	30,782	21	0.000682	0.00136	0.47
	Other ethnicities	6462	8	0.00124	0.00248	1.53
*FGB*	All	277,144	231	0.000833	0.00167	0.69
	Africans and African Americans	24,032	11	0.000458	0.000915	0.21
	Admixed Americans	34,418	39	0.00113	0.00226	1.28
	Ashkenazi Jewish	10,152	0	-	-	-
	East Asians	18,868	12	0.000636	0.00127	0.40
	Finnish	25,790	4	0.000155	0.000310	0.024
	Europeans (not Finnish)	126,646	137	0.00108	0.00216	1.17
	South Asians	30,782	23	0.000747	0.00149	0.56
	Other ethnicities	6466	5	0.000773	0.00155	0.60
*FGG*	All	277,080	856	0.00309	0.00618	9.54
	Africans and African Americans	24,034	64	0.00266	0.00532	7.09
	Admixed Americans	34,394	56	0.00163	0.00326	2.65
	Ashkenazi Jewish	10,150	13	0.00128	0.00256	1.64
	East Asians	18,854	13	0.000689	0.00138	0.47
	Finnish	25,790	40	0.00155	0.00310	2.40
	Europeans (not Finnish)	126,630	608	0.00480	0.00960	23.05
	South Asians	30,782	43	0.00140	0.00280	1.95
	Other ethnicities	6462	19	0.00294	0.00588	8.65

Carrier rates were estimated using the gnomAD database, including all null mutations, plus missense variants predicted to be deleterious by seven of seven prediction software, plus splicing defects located at intronic positions −3 and +3/+6 and predicted as disrupting by three of three algorithms (see Section 4). ^1^ Discrepancies in the number of alleles are due to slight differences in the number of individuals successfully sequenced for each specific region; ^2^ Mutations that were reported in fibrinogen-related databases as associated with dysfibrinogenemia, hypodysfibrinogenemia, and amyloidosis were not included.

**Table 3:** Estimated global prevalence of recessively-inherited fibrinogen deficiencies by ethnicity.

Population	Prevalence in 10^6^ Individuals
All	10.54
Africans and African Americans	7.44
Admixed Americans	5.35
Ashkenazi Jewish	1.64
East Asians	1.05
Finnish	2.42
Europeans (not Finnish)	24.5
South Asians	2.98
Other ethnicities	10.78

Carrier rates calculated for the three fibrinogen genes (Table 2) were summed to estimate global carrier rates in different populations.

**Table 4:** Most frequent mutations causing recessively-inherited fibrinogen deficiencies by ethnicity and gene.

Gene	Population	RefSeqID	Genomic Position on Chr 4 ^1^	Type of Mutation	Consequence ^2^	% of All Mutated Alleles in the Population ^3^	Allele Freq in gnomAD Database
*FGA*	Admixed Americans	rs773619297	155,506,927	frameshift	p.Gly552AlafsTer16	24.4	0.00029
		rs748106542	155,508,067	missense	p.Asp172Asn	29.3	0.00036
	Europeans (not Finnish)	rs773619297	155,506,927	frameshift	p.Gly552AlafsTer16	11.9	0.000063
		rs146387238	155,508,663	splice donor	c.510+1G>T *	17.9	0.000094
*FGB*	Admixed Americans	rs774502903	155,487,641	missense	p.Gly103Arg	41.0	0.00047
	Europeans (not Finnish)	rs370703973	155,489,611	missense	p.Tyr266Cys *	51.1	0.00055
		rs777451745	155,490,421	missense	p.Ala307Val *	10.2	0.00011
	South Asians	rs762523152	155,490,854	missense	p.Leu383Val	26.1	0.00019
		rs765571602	155,491,770	missense	p.Met482Val	34.8	0.00026
*FGG*	Africans and African Americans	rs145051028	155,529,735	missense	p.Ser245Phe	54.7	0.0015
		rs148685782	155,533,035	missense	**p.Ala108Gly** *	28.1	0.00075
	Admixed Americans	rs776288074	155,530,871	missense	p.Tyr193His	35.7	0.00058
		rs148685782	155,533,035	missense	**p.Ala108Gly** *	19.6	0.00032
		rs138511699	155,533,337	missense	p.Thr47Ile *	23.2	0.00038
	Ashkenazi Jewish	rs148685782	155,533,035	missense	**p.Ala108Gly** *	100	0.0013
	Finnish	rs148685782	155,533,035	missense	**p.Ala108Gly** *	72.5	0.0011
		rs138511699	155,533,337	missense	p.Thr47Ile *	27.5	0.00043
	Europeans (not Finnish)	rs148685782	155,533,035	missense	**p.Ala108Gly** *	75.0	0.0036
		rs138511699	155,533,337	missense	p.Thr47Ile *	15.0	0.00074
	South Asians	rs148685782	155,533,035	missense	**p.Ala108Gly** *	27.9	0.00039
		rs138511699	155,533,337	missense	p.Thr47Ile *	46.5	0.00066

^1^ Numbering according to UCSC Genome Browser, human, February 2009 (GRCh37/hg19) assembly. ^2^ The consequences on the native-protein numbering are reported. Mutations reported in fibrinogen-related databases (Human Fibrinogen Database, HGMD, ExPASy, ClinVar) are indicated with an asterisk. ^3^ Frequencies relative to the corresponding gene. The highly-recurrent *FGG* p.Ala108Gly and *FGG* p.Thr47Ile mutations are respectively depicted in bold and underlined.

**Table 5:** Estimated prevalence of autosomal-dominant fibrinogen disorders by ethnicity.

Population	Total Number of Alleles ^1^	Total Number of Variants	Collective Frequency of Variants	Heterozygote Frequency	Prevalence in 10^3^ Individuals
All	277,144	1524	0.0055	0.011	11
Africans and African Americans	24,036	166	0.0069	0.014	14
Admixed Americans	34,418	176	0.0051	0.010	10
Ashkenazi Jewish	10,152	44	0.0043	0.0087	9
East Asians	18,868	35	0.0019	0.0037	4
Finnish	25,794	44	0.0017	0.0034	3
Non-Finnish Europeans	126,646	922	0.0073	0.015	15
South Asians	30,782	90	0.0029	0.0058	6
Other ethnicities	6466	47	0.0073	0.015	15

Carrier rates were estimated using the gnomAD database, including all null mutations, plus missense variants predicted to be deleterious by 7 of 7 prediction software, plus splicing defects located at intronic positions −3 and +3/+6 and predicted as disrupting by 3 of 3 algorithms (see Section 4). ^1^ Discrepancies in the number of alleles are due to slight differences in the number of individuals successfully sequenced for each specific region.

**Table 6:** World-wide frequency of dysfibrinogenemia-, fibrinogen storage disease (FSD)-, and hereditary renal amyloidosis (HRA)-causing hotspot mutations.

Gene	Disorder	Mutation (Legacy Name)	Mutation (gnomAD)	Number of Mutated Alleles ^1^
*FGA*	dysfibrinogenemia	Aα(16)Arg > Cys	p.Arg35Cys	1
	dysfibrinogenemia	Aα(16)Arg > His	p.Arg35His	5
	HRA	Aα(526)Glu > Lys	p.Glu545Lys	29
	HRA	Aα(526)Glu > Val	p.Glu545Val	1
*FGG*	dysfibrinogenemia	γ(275)Arg > His	p.Arg301His	2

Hotspot mutations were searched in the gnomAD database. ^1^ The overall world-wide population was considered (277,144 total alleles).

**Table 7:** Composition of the gnomAD database.

Population	Number of Genomes	Number of Exomes	Total
African/African American	4368	7652	12,020
Admixed American	419	16,791	17,210
Ashkenazi Jewish	151	4925	5076
East Asian	811	8624	9435
Finnish	1747	11,150	12,897
Non-Finnish European	7509	55,860	63,369
South Asian	0	15,391	15,391
Other (population not assigned)	491	2743	3234
Total	15,496	123,136	138,632

## Supplementary Materials

Supplementary materials can be found at www.mdpi.com/1422-0067/18/12/2711/s1.

## Abbreviations

ER	Endoplasmic Reticulum
Aα	A α Polypeptide Chain
Bβ	B β Polypeptide Chain
γ	γ Polypeptide chain
*FGA*	Fibrinogen α Chain Gene
*FGB*	Fibrinogen β Chain Gene
*FGG*	Fibrinogen γ Chain Gene
OMIM	Online Mendelian Inheritance in Man
FSD	Fibrinogen Storage Disease
HRA	Hereditary Renal Amyloidosis
gnomAD	Genome Aggregation Database
HGMD	Human Gene Mutation Database
ExPASy	Expert Protein Analysis System
WFH	World Federation of Hemophilia
LRT	Likelihood Ratio Test
FATHMM	Functional Analysis Through Hidden Markov Model
