# Supplementary material for: Fibrinogen as a Pleiotropic Protein Causing Human Diseases: The Mutational Burden of Aα, Bβ, and γ Chains
Source: Int J Mol Sci. 2017 Dec 14;18(12):2711. doi: 10.3390/ijms18122711 (PMC5751312; doi:10.3390/ijms18122711)
Supplement: Supplementary file 1 [file ijms-18-02711-s001.pdf]

Supplementary Material

Article

Fibrinogen as a Pleiotropic Protein Causing Human Diseases: The Mutational Burden of Aα, Bβ, and γ Chains

Elvezia Maria Paraboschi [1], Stefano Duga [1,2] and Rosanna Asselta [1,2,*]

[1] Department of Biomedical Sciences, Humanitas University, Via Rita Levi Montalcini 4, 20090 Pieve Emanuele, Milan, Italy; elvezia_maria.paraboschi@hunimed.eu (E.M.P.); stefano.duga@hunimed.eu (S.D.)
[2] Humanitas Clinical and Research Center, Via Manzoni 56, 20089 Rozzano, Milan, Italy
* Correspondence: rosanna.asselta@hunimed.eu; Tel.: +39-2-8224-5215

List of contents:

- Supplementary table 1: Null frameshift and nonsense mutations present in the gnomAD database.
- Supplementary table 2: Missense/splicing/inframe-indels mutations consistently predicted as dangerous by prediction programs.
- Supplementary table 3. Mutational burden of the fibrinogen gene cluster in the Italian population.
- Supplementary Table 4. Estimated prevalence of autosomal-dominant fibrinogen disorders by ethnicity excluding the frequent p.Ala108Gly mutation.

Supplementary Table 1. Null frameshift and nonsense mutations present in the gnomAD database.

Gene	RefSeqID	Genomic position [1]	cDNA level	Native protein
FGA	-	Chr4:155,510,678	c.91G>T	p.Gly31Ter
	-	Chr4:155,510,591	c.177delC	p.Trp60GlyfsTer11
	rs767657961	Chr4:155,510,037	c.272C>A	p.Ser91Ter
	-	Chr4:155,509,956	c.352delT	p.Ser118ProfsTer17
	-	Chr4:155,508,799	c.374delA	p.Asn125IlefsTer10
	rs770680765	Chr4:155,508,686	c.488T>A	p.Leu163Ter
	rs764043296	Chr4:155,507,999	c.581dupT	p.Lys195GlufsTer4
	rs776817952	Chr4:155,507,659	c.922C>T	p.Arg308Ter
	rs752823417	Chr4:155,507,490	c.1086_1090delTGGCA	p.Gly363LeufsTer2
	-	Chr4:155,507,428	c.1153C>T	p.Gln385Ter
	rs767571876	Chr4:155,507,362	c.1215_1218dupTAAC	p.Asn407Ter
	rs759819642	Chr4:155,507,299	c.1280_1281dupAG	p.Tyr428SerfsTer57
	rs774564301	Chr4:155,507,283	c.1288_1297delACAGAAAAAC	p.Thr430TrpfsTer51
	-	Chr4:155,507,054	c.1526dupT	p.Arg510ProfsTer3
	rs4766	Chr4:155,507,039	c.1541delC	p.Pro514LeufsTer24
	-	Chr4:155,506,953	c.1626_1627delTG	p.Ser542ArgfsTer3
	rs773619297	Chr4:155,506,927	c.1653delT	p.Gly552AlafsTer16
	-	Chr4:155,506,917	c.1663dupA	p.Thr555AsnfsTer15
	rs750277272	Chr4:155,506,851	c.1730C>G	p.Ser577Ter
	rs770461175	Chr4:155,506,844	c.1736dupA	p.Tyr579Ter
	-	Chr4:155,506,693	c.1888A>T	p.Arg630Ter
FGB	-	Chr4:155,487,776	c.443delT	p.Met148SerfsTer4
	-	Chr4:155,488,826	c.575delC	p.Pro192GlnfsTer14
	-	Chr4:155,488,923	c.669T>A	p.Cys223Ter
	rs775981072	Chr4:155,490,357	c.856C>T	p.Gln286Ter
	-	Chr4:155,490,754	c.1048delG	p.Asp350ThrfsTer3
	rs547765642	Chr4:155,490,757	c.1055_1059delTAAAG	p.Val352GlyfsTer11
FGG	rs778213856	Chr4:155,533,224	c.253C>T	p.Gln85Ter
	-	Chr4:155,533,022	c.335_336insTCAACATAAT	p.Arg113GlnfsTer14
	-	Chr4:155,532,999	c.358delA	p.Met120Ter
	rs750923281	Chr4:155,530,878	c.568_569delAG	p.Ser190ArgfsTer6
	rs768694513	Chr4:155,530,797	c.651G>A	p.Trp217Ter
	-	Chr4:155,529,686	c.782dupT	p.Leu261PhefsTer36
	-	Chr4:155,528,073	c.906_912dupAACATAT	p.Ala305AsnfsTer15
	-	Chr4:155,527,860	c.1126C>T	p.Gln376Ter

These mutations appear in the gnomAD database in at least 1 individual; none of them has been described in fibrinogen-related databases. [1]Numbering according to UCSC Genome browser, human assembly, February 2009 (GRCh37/hg19).

Supplementary Table 2. Missense/splicing/inframe-indels mutations consistently predicted as dangerous by prediction programs.

Gene	RefSeqID	Genomic position [1]	cDNA level	Native protein
FGA	rs151016432	Chr4:155,508,036	c.545G>C	p.Gly182Ala
	rs754195439	Chr4:155,508,048	c.533G>C	p.Arg178Pro
	rs754195439	Chr4:155,508,048	c.533G>A	p.Arg178Gln
	-	Chr4:155,508053	c.528G>C	p.Lys176Asn
	-	Chr4:155,508,054	c.527A>T	p.Lys176Met
	rs780068895	Chr4:155,508,065	c.516C>G	p.Asp172Glu
	rs748106542	Chr4:155,508,067	c.514G>A	p.Asp172Asn
	rs780339572	Chr4:155,508,665	c.509A>T	p.Glu170Val
	-	Chr4:155,508,666	c.508G>C	p.Glu170Gln
	rs771565963	Chr4:155,508,706	c.468G>T	p.Gln156His
	rs765688392	Chr4:155,508,740	c.434T>C	p.Val145Ala
	rs147888314	Chr4:155,508,771	c.403A>G	p.Arg135Gly
	rs747854049	Chr4:155,510,070	c.239A>G	p.Asp80Gly
	-	Chr4:155,510,071	c.238G>T	p.Asp80Tyr
	rs749744029	Chr4:155,510,095	c.214G>C	p.Gly72Arg
	-	Chr4:155,510,115	c.194C>A	p.Pro65His
	-	Chr4:155,510,593	c.176A>G	p.Asp59Gly
	rs765285466	Chr4:155,510,599	c.170A>G	p.Asp57Gly
	-	Chr4:155,510,615	c.154T>C	p.Trp52Arg
	-	Chr4:155,510,615	c.154T>A	p.Trp52Arg
	rs753733924	Chr4:155,509,937	c.364+4_364+7delAGTA	-
	rs752713439	Chr4:155,509,941	c.364+4A>G	-
FGB	rs765627047	Chr4:155,487,117	c.272A>T	p.Asp91Val
	rs774502903	Chr4:155,487,641	c.307G>A	p.Gly103Arg
	-	Chr4:155,487,648	c.314T>C	p.Leu105Ser
	rs779413540	Chr4:155,487,669	c.335T>C	p.Leu112Ser
	rs530036524	Chr4:155,488,841	c.587G>A	p.Arg196His
	-	Chr4:155,488,843	c.589G>A	p.Val197Met
	rs747314723	Chr4:155,488,849	c.595C>T	p.Arg199Cys
	-	Chr4:155,488,799	c.545A>G	p.Tyr182Cys
	rs548206997	Chr4:155,488,850	c.596G>A	p.Arg199His
	rs758583316	Chr4:155,488,878	c.624A>C	p.Lys208Asn
	rs538708636	Chr4:155,488,880	c.626T>C	p.Ile209Thr
	rs765701687	Chr4:155,488,952	c.698T>G	p.Ile233Ser
	rs750327950	Chr4:155,489,578	c.764A>G	p.Tyr255Cys
	rs758467916	Chr4:155,489,584	c.770T>C	p.Ile257Thr
	-	Chr4:155,489,622	c.808T>C	p.Cys270Arg
	rs771406104	Chr4:155,490,343	c.842T>C	p.Val281Ala
	-	Chr4:155,490,347	c.846T>G	p.Ile282Met
	rs368867072	Chr4:155,490,686	c.979G>T	p.Asp327Tyr
	rs766420576	Chr4:155,490,732	c.1025T>C	p.Ile342Thr
	rs751610009	Chr4:155,490,733	c.1026A>G	p.Ile342Met
	rs150330219	Chr4:155,490,738	c.1031T>C	p.Met344Thr
	rs761339656	Chr4:155,490,752	c.1045G>A	p.Gly349Arg
	rs764989450	Chr4:155,490,753	c.1046G>C	p.Gly349Ala
	-	Chr4:155,490,782	c.1075T>C	p.Phe359Leu
	rs764970021	Chr4:155,490,845	c.1138G>A	p.Gly380Ser
	rs762523152	Chr4:155,490,854	c.1147C>G	p.Leu383Val
	rs754035448	Chr4:155,490,906	c.1199A>C	p.His400Pro

	-	Chr4:155,490,912	c.1205G>T	p.Gly402Val
	-	Chr4:155,490,933	c.1226A>G	p.Asp409Gly
	rs776578431	Chr4:155,490,941	c.1234A>T	p.Asn412Tyr
	-	Chr4:155,490,942	c.1235A>G	p.Asn412Ser
	-	Chr4:155,491,623	c.1297T>A	p.Trp433Arg
	rs764555923	Chr4:155,491,635	c.1309T>C	p.Cys437Arg
	rs765571602	Chr4:155,491,770	c.1444A>G	p.Met482Val
	rs769690072	Chr4:155,491,568	c.1245-3C>G	-
FGG	rs746473186	Chr4:155,526,080	c.1265_1267delAAG	p.Glu422del
	rs776109029	Chr4:155,526,108	c.1240C>A	p.Pro414Thr
	-	Chr4:155,527,954	c.1032C>A	p.Asp344Glu
	rs746474853	Chr4:155,528,014	c.972T>A	p.Asp324Glu
	rs777248925	Chr4:155,528,022	c.964G>A	p.Gly322Ser
	rs765095457	Chr4:155,528,106	c.880G>A	p.Gly294Arg
	rs750162680	Chr4:155,528,109	c.877G>A	p.Val293Met
	rs376120251	Chr4:155,529,690	c.779A>G	p.His260Arg
	rs757602733	Chr4:155,529,699	c.770A>C	p.Glu257Ala
	-	Chr4:155,529,717	c.752A>G	p.Glu251Gly
	-	Chr4:155,529,720	c.749C>T	p.Thr250Ile
	rs145051028	Chr4:155,529,735	c.734C>T	p.Ser245Phe
	rs372737021	Chr4:155,529,789	c.680G>A	p.Ser227Asn
	rs758757675	Chr4:155,530,784	c.664A>G	p.Lys222Glu
	rs747416023	Chr4:155,530,795	c.653C>T	p.Thr218Ile
	rs781597393	Chr4:155,530,799	c.649T>G	p.Trp217Gly
	rs771885312	Chr4:155,530,823	c.625G>A	p.Glu209Lys
	rs776288074	Chr4:155,530,871	c.577T>C	p.Tyr193His
	-	Chr4:155,530,879	c.569G>T	p.Ser190Ile
	rs868267949	Chr4:155,530,912	c.536G>T	p.Cys179Phe
	rs371692602	Chr4:155,531,248	c.503C>T	p.Thr168Met
	-	Chr4:155,531,344	c.407T>G	p.Leu136Trp
	-	Chr4:155,533,331	c.146G>C	p.Cys49Ser
	-	Chr4:155,533,334	c.143C>T	p.Thr48Ile
	rs202132393	Chr4:155,533,353	c.124G>T	p.Gly42Cys
	-	Chr4:155,533,566	c.100T>G	p.Cys34Gly
	-	Chr4:155,532,952	c.401+5G>A	-
	rs556828126	Chr4:155,533,680	c.78+4A>G	-

These mutations appear in the gnomAD database in at least 1 individual; none of them has been described in fibrinogen-related databases; they were predicted as deleterious by 7 of 7 prediction software (in the case of missense or in-frame indels) or 3 of 3 (in case of splicing mutations). [1]Numbering according to UCSC Genome browser, human assembly, February 2009 (GRCh37/hg19).

Supplementary Table 3. Mutational burden of the fibrinogen gene cluster in the Italian population.

Gene (prevalence)	RefSeqID	Genomic position [1]	Native protein	Number of Italians carrying the identified mutation	Present in fibrinogen-related databases
FGA (0.082)	-	155,510,651	p.Val40TrpfsTer31	1	a
FGB (0.33)	-	155,490,343	p.Val281Ala	1	-
	-	155,490,752	p.Gly349Arg	1	-
FGG (36)	-	155,529,674	p.Gln265His	2	d
	-	155,529,760	p.Tyr237His	1	h
	-	155,530,795	p.Thr218Ile	1	-
	-	155,531,248	p.Thr168Met	2	-
	rs148685782	155,533,035	p.Ala108Gly	15	h
	rs138511699	155,533,337	p.Thr47Ile	2	h
	-	155,533,353	p.Gly42Ser	5	d

The prevalence of recessively-inherited fibrinogen disorders is reported in brackets below the relevant gene (for million people); the cumulative prevalence corresponds to 36.4 in 10^6; for these calculations, mutations reported in fibrinogen-related databases as cause of dysfibrinogenemia (d) or amyloidosis (m) were not included; a, afibrinogenemia; h, hypofibrinogenemia. Missense variants not reported in fibrinogen-related databases were predicted as deleterious by 7 of 7 prediction software. [1]Numbering according to UCSC Genome browser, human assembly, February 2009 (GRCh37/hg19).

Supplementary Table 4. Estimated prevalence of autosomal-dominant fibrinogen disorders by ethnicity excluding the frequent p.Ala108Gly mutation.

Population	Total number of alleles [1]	Total number of variants	Collective frequency of variants	Heterozygote frequency	Prevalence in 10^3 individuals
**ALL**	277,144	826	0.0030	0.0060	6
Africans and African Americans	24,036	134	0.0056	0.011	11
Admixed Americans	34,418	136	0.0040	0.0080	8
Ashkenazi Jewish	10,152	19	0.0019	0.0037	4
East Asians	18,868	33	0.0017	0.0035	3
Finnish	25,794	15	0.0006	0.0012	1
Non-Finnish Europeans	126,646	389	0,0031	0.0060	6
South Asians	30,782	78	0.0025	0.0051	5
Other ethnicities	6,466	22	0.0034	0.0068	7

Carrier rates were estimated using the gnomAD database, including all null mutations, plus missense variants predicted to be deleterious by 7 of 7 prediction software, plus splicing defects located at intronic positions -3 and +3/+6 and predicted as disrupting by 3 of 3 algorithms (see Materials and Methods). The p.Ala108Gly mutation has been excluded from this analysis. [1]Discrepancies in the number of alleles are due to slight differences in the number of individuals successfully sequenced for each specific region.
